# Saphenous Vein Graft Pseudoaneurysm Treated With Covered Stenting

**DOI:** 10.7759/cureus.64686

**Published:** 2024-07-16

**Authors:** Jay N Patel, David R Anderson, Muhammad S Ghauri, Joseph Yang, Jeffrey Zimmet, Elaine Tseng, Kendrick A Shunk, Marko Boskovski

**Affiliations:** 1 Cardiothoracic Surgery, California University of Science and Medicine, Colton, USA; 2 Interventional Cardiology, Kaiser Permanente North Valley, Roseville, USA; 3 Neurosurgery, California University of Science and Medicine, Colton, USA; 4 Interventional Cardiology, University of California, San Francisco Medical Center, San Francisco, USA; 5 Cardiothoracic Surgery, San Francisco Veterans Affairs Health Care System, San Francisco, USA; 6 Interventional Cardiology, San Francisco Veterans Affairs Health Care System, San Francisco, USA

**Keywords:** endovascular procedure, coronary artery bypass grafting, coronary angiography, percutaneous coronary intervention, saphenous vein graft pseudoaneurysm

## Abstract

Saphenous vein graft (SVG) pseudoaneurysms are an infrequent, but life-threatening complication of coronary artery bypass grafting (CABG) surgery if left untreated. Here, we discuss the case of a 77-year-old patient, with a prior history of CABG and transcatheter aortic valve implantation (TAVI), who was incidentally found on computed tomography angiography (CTA) to have a pseudoaneurysm of his SVG with an initial chief complaint of dizziness. Despite increasing reports of SVG pseudoaneurysm, there is no consensus on definitive treatment. Due to the high mortality risk of this patient with surgical intervention, a minimally invasive percutaneous coronary intervention was performed. The patient was effectively treated with two overlapping Viabahn-covered stents, which completely excluded the pseudoaneurysm. Follow-up imaging at two months showed two well-positioned overlapping self-expanding stents with total occlusion of the pseudoaneurysm.

## Introduction

Myocardial infarction (MI) is a leading cause of morbidity and mortality in the developed world [1]. In the United States, MIs pose a significant economic burden costing an estimated 84.9 billion dollars [2]. Percutaneous coronary intervention (PCI) revascularization using bare metal or drug-eluting stents is widely used for patients with confirmed MI. However, in patients with complex multivessel disease or pathology affecting the left main coronary artery, coronary artery bypass graft (CABG) surgery is the gold-standard treatment [1]. The left internal mammary artery (LIMA) remains the predominant graft source for diseased vessels in conjunction with saphenous vein grafts (SVGs) for multilevel disease [3].

Despite increasing understanding and advancements in surgical techniques and adjunctive pharmacotherapy, SVG failure rates remain high, predisposing patients to potential repeat coronary revascularizations [3]. SVG failure following CABG is commonly due to diameter size mismatch, which results in non-laminar flow and can impact graft patency [4]. Clinically, SVG failure can manifest with pathologic conduction abnormalities on electrocardiograms (EKGs) or present insidiously and found incidentally on imaging. Commonly employed techniques to minimize endothelial damage include the no-touch SVG technique, which minimizes graft manipulation to reduce the risk of graft failure [3]. In addition, optimizing revascularization strategies through the careful selection of target vessels, appropriate graft lengths, and ensuring adequate flow and minimizing resistance at anastomotic sites via transit time flow measurements [1,3,5] to reduce the incidence of SVG failure. Despite these strategies, SVG failure rates can be as high as 12% prior to discharge from the hospital, up to 25% by the end of the first year, and up to 60% of grafts remaining patent after 10 years [3].

The pathophysiologic mechanisms leading to SVG failure have been well studied, which include thrombosis and technical failure, intimal hyperplasia, and atherosclerosis [3]. Early complications of saphenous venous graft failure in CABG include graft occlusion, embolism, graft infection, cardiac conduction system abnormalities, and cardiac arrest [6]. Late complications of CABG include SVG aneurysms and pseudoaneurysms, for which aneurysmal rupture usually occurs within five years after surgery [7]. However, SVG pseudoaneurysms are rare complications after CABG with only less than 5% of patients developing them [8].

Currently, the management of SVG failure due to pseudoaneurysms includes redo-surgery, PCI (including vascular plugs, covered stents, and embolization coils), and conservative management [9]. Considering that there is no consensus guiding definitive management, the treatment modality is usually selected on a case-by-case basis [10]. Here, we describe a unique case of a large SVG pseudoaneurysm that was successfully managed via PCI with covered stent placement.

## Case presentation

An asymptomatic 77-year-old man with a history of a CABG in 1985 and transcatheter aortic valve implantation (TAVI) in 2020 was referred to the San Francisco Veterans Affairs Medical Center for evaluation of an incidentally found 5.7 cm pseudoaneurysm of his SVG to the right coronary artery (RCA). The pseudoaneurysm was discovered on a head, neck, and chest computed tomography angiography (CTA) performed as part of a workup for dizziness months after the TAVI procedure.

The patient has a past medical history of paroxysmal atrial fibrillation, hypertension, hyperlipidemia, and coronary artery disease. He is a former smoker with a 30-pack-year history and mild chronic obstructive pulmonary disease (COPD). In 1985, he underwent a two-vessel CABG with an SVG to the LAD and RCA. He had a TAVI completed in 2020 for symptomatic severe aortic stenosis and subsequently required a permanent pacemaker for a complete heart block.

**Figure 1:**
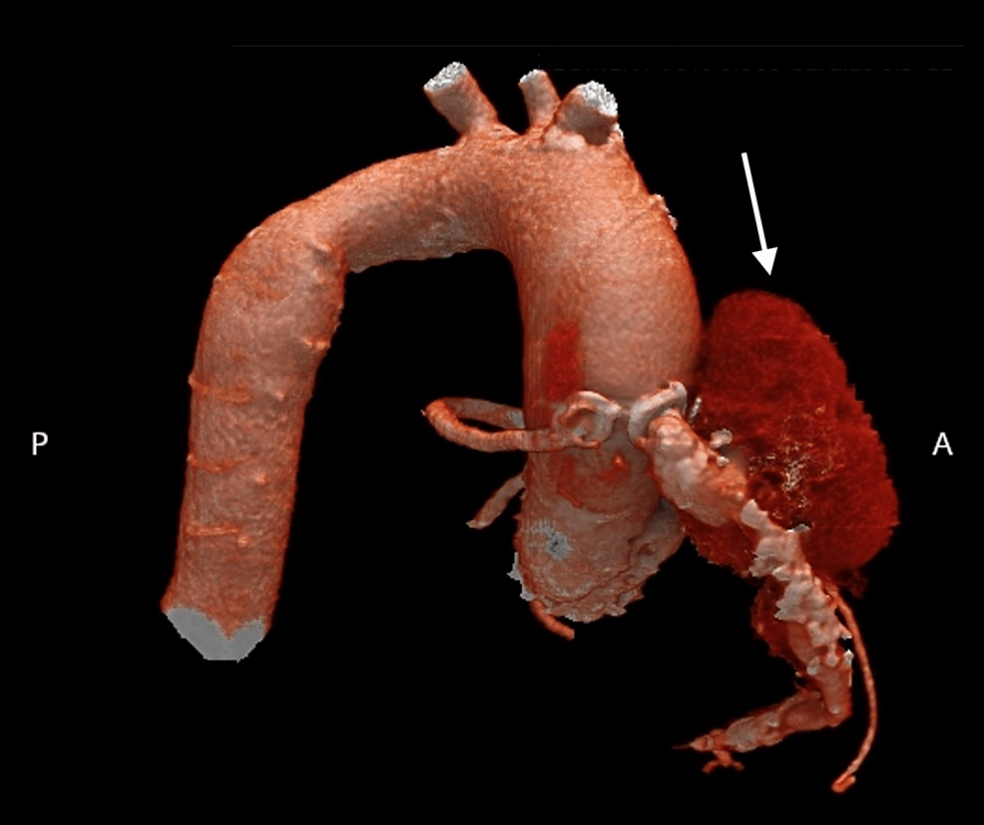
Pre-Stent deployment CT 3D volume-rendered imaging Computed tomography three-dimensional volume render scan showing a pseudoaneurysm in the saphenous vein graft (SVG) to the right coronary artery (RCA), measuring 5.7 cm in size.

**Figure 2:**
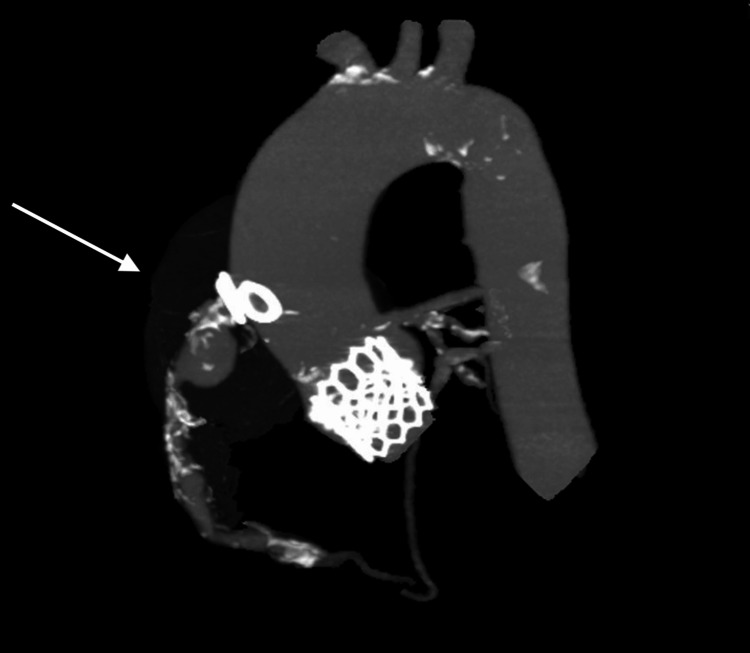
Pre-stent CT image highlighting SVG-RCA pseudoaneurysm Computed tomography scan showing a saphenous vein graft (SVG) to right coronary artery (RCA) pseudoaneurysm. The aortic-ostia diameter by the CT measured ~6.5 x 6 mm. A total length of 150 mm from the proximal to distal end of the pseudo-aneurysmal segment was needed to effectively cover and seal the pseudoaneurysm.

A gated cardiac coronary CTA with three-dimensional reconstruction was used to evaluate the coronary anatomy including the SVG to RCA pseudoaneurysm. Post-processing 3D reconstructions revealed that the largest diameter of the pseudoaneurysm was 5.7 cm (Figure 1). In addition, the SVG-RCA aortic-ostia junctional diameter by computed tomography (CT) measured ~6 x 6.5 mm and the approximate length of the lesion needed to exclude the entire pseudoaneurysm measured ~150 mm, from the ostia to a healthy distal reference vessel (Figure 2). ECG showed atrial fibrillation with AV dual-paced rhythm. Transthoracic echocardiogram showed normal left (LVEF 60%) and right ventricular function, as well as a 26 mm balloon expandable aortic prosthesis with a mean gradient of 15 mmHg and mild-moderate regurgitation. There was mild left atrial enlargement with no other significant valvular disease.

The patient’s calculated Society of Thoracic Surgeons (STS) risk of mortality with surgical coronary re-intervention was 4.2%, and his overall functional status was limited. After a heart team conference, we decided to pursue percutaneous intervention. Similar to the endovascular treatment approach of aneurysms and pseudoaneurysms in other parts of the body, we planned to exclude the pseudoaneurysm with off-label covered stents anchored in the proximal (ostial) and distal aspect of the vein graft, thereby preventing flow in and around the edges of the stent. The stent size and length were chosen based on the CTA 3D reconstruction vein graft (Figure 2). The left brachial artery was accessed to allow easy reach of the SVG with a multipurpose guide and destination sheath. Using a 0.018’ wire in the distal vessel and a destination sheath deep in the vein graft, we deployed two overlapping Viabahn stents, the first being 7 x 100 mm and the second being 8 x 75 mm, which effectively covered the pseudoaneurysm, as seen in the angiogram (see Videos 1, 2). Follow-up imaging at two months showed two well-positioned overlapping self-expanding stents with total occlusion of the pseudoaneurysm.

**Video 1:** Coronary angiogram showing SVG-RCA pseudoaneurysm Coronary angiography showing a 0.018’ wire in the distal vessel and a destination sheath deep in the vein graft prior to deploying 2 Viabahn stents in the SVG pseudoaneurysm.

**Video 2:** Coronary angiogram showing overlapping 7 x 100 mm and 8 x 75 mm Viabahn stents excluding the SVG pseudoaneurysm to the ostia Coronary angiography showing a 0.018’ wire in the distal vessel and a destination sheath deep in the vein graft after deploying overlapping Viabahn stents in the SVG pseudoaneurysm. The first was 7 x 100 mm and the second was 8 x 75 mm.

## Discussion

SVG pseudoaneurysms are an infrequent, but life-threatening complication of CABG that can cause significant morbidity and mortality if left untreated [11-12]. Unlike aneurysms, which involve the weakening of all three layers of the blood vessel, pseudoaneurysms result from the disruption of the intima and media, with resultant hematoma formation that is contained by the adventitia or surrounding structures. Aneurysms commonly form in the body of the graft, whereas pseudoaneurysms usually occur at the anastomotic sites [4]. They may arise because of surgical complications, such as weak anastomotic suture lines, infections along the graft, or mechanical trauma/shear force [1,12]. These pseudoaneurysms most commonly occur in the RCA grafts, followed by left circumflex coronary artery grafts, and lastly by the left anterior artery grafts [13]. The differential location of pseudoaneurysm formation could be attributed to varying difficulties of performing an optimal graft anastomosis depending on coronary artery location after heart manipulation for the target vessel. Early detection of these by CT or magnetic resonance imaging in patients with a high clinical suspicion of SVG pseudoaneurysms is necessary as most patients remain asymptomatic at presentation or have an unrelated chief complaint [13]. Failure to detect SVG pseudoaneurysms can result in adverse events, such as pseudoaneurysm rupture, extrinsic compression of nearby structures, fistula formation, and death [1,10,13]. Treatment options for SVG aneurysms include surgery, PCI (including vascular plugs, covered stents, and embolization coils), and conservative management [9]. Although the first-line treatment for SVG pseudoaneurysms utilizes surgical repair for definitive repair, a percutaneous approach may be implemented as a less invasive treatment option [11].

Despite the rarity of SVG pseudoaneurysms, they have been increasingly reported over the past few years [13]. Smer et al. performed a systematic review of 72 cases of SVG pseudoaneurysm revealing varying case presentations, ranging from asymptomatic or mild symptoms to life-threatening emergencies, including rupture, fistula formation, cardiac tamponade, and compression of adjacent cardiac structures [13]. In addition, compared to the STS-reported re-exploration rate of 3.1% among CABGs, 22% of all patients with SVG pseudoaneurysms required postoperative surgical re-exploration. Furthermore, 14 out of 72 (19.4%) cases utilized covered stents, but the limited and variable short- and long-term follow-up data significantly limited outcome interpretation [13]. Dieter et al. performed a retrospective cohort analysis of 13 patients and found that compared with conservative management, early surgical treatment of SVG aneurysms failed to improve survival [14].

Currently, there is no consensus in the medical community on a standard treatment approach therefore patients should undergo appropriate risk stratification to determine the best individualized treatment strategy [11]. Considering that there are no randomized trials comparing the efficacy of these treatment options, we opted for a percutaneous intervention given the high surgical risk of our patient. Our treatment approach involved meticulous preoperative planning using 3D reconstruction imaging and a multidisciplinary team execution. We successfully performed percutaneous exclusion of an SVG to RCA pseudoaneurysm using two overlapping covered stents. To our knowledge, there have been only a few reported cases in the literature where a covered stent was implemented to effectively treat SVG pseudoaneurysms [1,13,15-16]. Although there are also no recommendations on follow-up imaging, we elected to evaluate the repair on the first post-procedural day and after two months. Our patient had no complications and demonstrated a complete resolution of his presenting symptoms. Our approach contributes to the continued understanding of minimally invasive techniques to treat SVG pseudoaneurysms in high-risk patients.

## Conclusions

SVG pseudoaneurysms are rare, but serious complications of CABG. Although there is no consensus in the medical community on a standard treatment approach, percutaneous and surgical strategies are available, and each patient should be evaluated individually. Here, we successfully performed percutaneous exclusion of an SVG to RCA pseudoaneurysm using two overlapping covered stents. These results confirm previously reported successfully treated SVG pseudoaneurysms with covered stents. Prospective studies with long-term follow-up are necessary to evaluate the outcomes of patients with these rare complications.
